# Prostate adenocarcinoma and COVID‐19: The possible impacts of *TMPRSS2* expressions in susceptibility to SARS‐CoV‐2

**DOI:** 10.1111/jcmm.16385

**Published:** 2021-02-20

**Authors:** Jingliang Cheng, Ju Zhou, Shangyi Fu, Jiewen Fu, Baixu Zhou, Hanchun Chen, Junjiang Fu, Chunli Wei

**Affiliations:** ^1^ Key Laboratory of Epigenetics and Oncology the Research Center for Preclinical Medicine Southwest Medical University Luzhou China; ^2^ Department of Medical Technology Faculty of Associated Medical Sciences Chiang Mai University Chiang Mai Thailand; ^3^ Human Genome Sequencing Center Baylor College of Medicine Houston TX USA; ^4^ School of Medicine Baylor College of Medicine Houston TX USA; ^5^ Department of Gynecology and Obstetrics Guangdong Women and Children Hospital Guangzhou China; ^6^ Department of Biochemistry School of Life Sciences Central South University Changsha China

**Keywords:** COVID‐19, prostate adenocarcinoma, SARS‐CoV‐2, susceptibility, *TMPRSS2* gene

## Abstract

TMPRSS2 (OMIM: 602060) is a cellular protease involved in many physiological and pathological processes, and it facilitates entry of viruses such as SARS‐CoV‐2 into host cells. It is important to predict the prostate's susceptibility to SARS‐CoV‐2 infection in cancer patients and the disease outcome by assessing TMPRSS2 expression in cancer tissues. In this study, we conducted the expression profiles of the *TMPRSS2* gene for COVID‐19 in different normal tissues and PRAD (prostate adenocarcinoma) tumour tissues. TMPRSS2 is highly expressed in normal tissues including the small intestine, prostate, pancreas, salivary gland, colon, stomach, seminal vesicle and lung, and is increased in PRAD tissues, indicating that SARS‐CoV‐2 might attack not only the lungs and other normal organs, but also in PRAD cancer tissues. Hypomethylation of *TMPRSS2* promoter may not be the mechanism for TMPRSS2 overexpression in PRAD tissues and PRAD pathogenesis. TMPRSS2 expresses eleven isoforms in PRAD tissues, with the TMPRSS2‐001 isoform expressed highest and followed by TMPRSS2‐201. Further isoform structures prediction showed that these two highly expressed isoforms have both SRCR_2 and Trypsin (Tryp_SPc) domains, which may be essential for TMPRSS2 functional roles for tumorigenesis and entry for SARS‐CoV‐2 in PRAD patients. Analyses of functional annotation and enrichment in TMPRSS2 showed that TMPRSS2 is mostly enriched in regulation of viral entry into host cells, protein processing and serine‐type peptidase activity. TMPRSS2 is also associated with prostate gland cancer cell expression, different complex(es) formation, human influenza and carcinoma, pathways in prostate cancer, influenza A, and transcriptional misregulation in cancer. Altogether, even though high expression of TMPRSS2 may not be favourable for PRAD patient's survival, increased expression in these patients should play roles in susceptibility of the SARS‐CoV‐2 infection and clinical severity for COVID‐19, highlighting the value of protective actions of PRAD cases by targeting or androgen‐mediated therapeutic strategies in the COVID‐19 pandemic.

## 1 INTRODUCTION

Transmembrane serine protease 2 (TMPRSS2, OMIM: 602060), cytogenetic located at 21q22.3, was first identified by exon trapping in 1997, which encodes a 492 amino acids multimeric protein with a molecular mass 53 859 Da containing a serine protease domain.^1^ In prostate cancer tissues, Tomlins *et al* in 2005 identified recurrent gene fusions at the TMPRSS2 5′ UTR (untranslated region) to ETV1 or ERG with an outlier expression that drives cancer progression, suggesting oncogenic roles in prostate cancer.^2^ The cellular protease TMPRSS2 protein is highly expressed in secretory epithelial cells of the prostate, and its expression is androgen‐induced. As a member of serine protease family, TMPRSS2 is involved in many pathological and physiological processes,^3^, ^4^, ^5^ and also facilitates entry of viruses, including the human coronaviruses SARS‐CoV‐2 (severe acute respiratory syndrome coronavirus 2), SARS‐CoV (severe acute respiratory syndrome coronavirus), HCoV‐229E (human coronavirus‐229E), and MERS‐CoV (Middle East respiratory syndrome coronavirus), into host cells by cleaving and activating viral envelope glycoproteins, or proteolytical cleaving ACE2 (angiotensin‐converting enzyme 2) receptor (OMIM: 300332) for viral uptake.^6^, ^7^, ^8^, ^9^

Since December 2019, the coronavirus disease 2019 (COVID‐19) has rapidly spread worldwide, caused a global threat and the number of cases is rising worldwide.^10^, ^11^, ^12^, ^13^, ^14^ On 11 March 2020, the WHO (World Health Organization) declared COVID‐19 a global pandemic.^15^ At the end of December of 2020, the global confirmed cases are approximately 80 million and global deaths cases are nearly 2 million (https://coronavirus.jhu.edu/) worldwide. In addition to ACE2 as the SARS‐CoV‐2 virus enter receptor, Hoffmann et al recently revealed that the viral spike protein (S) is primed by TMPRSS2; thus, inhibitors of TMPRSS2 could block viral entry.^7^ This implies that suppression of the TMPRSS2 expression levels in normal cells might help fight not only prostate cancer developments but also the viral infection.

TMPRSS2 has an important role in the pathogenesis of COVID‐19, and the abnormal expression of *TMPRSS2* or *ERG* gene fusion is significant regulators of carcinogenesis in prostate cancer.^16^, ^17^, ^18^ By these reasons, it is important to predict the cancer patients’ susceptibility to SARS‐CoV‐2 infection and the disease outcome via assessing TMPRSS2 expression in cancer tissues, particular in prostate cancer tissues and related bioinformatics analyses. Thus, in this study, we performed the expression profile analyses of the *TMPRSS2* gene for COVID‐19 in different normal tissues and PRAD (prostate adenocarcinoma) tumour tissues as a marker for targeted therapy.^19^, ^20^, ^21^

## 2 MATERIALS AND METHODS

### 2.1 Homology analysis

Homologs of TMPRSS2 in humans (NP_001128571.1 in protein and NM_001135099.1 in gene from GenBank) and others from the NCBI program (https://www.ncbi.nlm.nih.gov/) were described previously.^22^

### 2.2 Expression analysis and databases

The expression levels of the human *TMPRSS2* gene in the normal tissues were assessed from the database, the Human Protein Atlas (HPA) (https://www.proteinatlas.org/ENSG00000184012‐TMPRSS2/tissue).^23^, ^24^, ^25^ The expression levels of *TMPRSS2* in the prostate adenocarcinoma (PRAD) and corresponding normal control tissues from TCGA‐PRAD (The Cancer Genome Atlas‐prostate adenocarcinoma) were evaluated via GEPIA 2 (The Gene Expression Profiling Interactive Analysis) (http://gepia2.cancer‐pku.cn/#analysis).^26^

### 2.3 Isoform analysis

By using GEPIA2,^26^ we explored the large TCGA (The Cancer Genome Atlas) and GTEx (Genotype‐Tissue Expression) datasets to determine TMPRSS2 isoform usage, expression distribution and domain structures (http://gepia2.cancer‐pku.cn/#isoform).

### 2.4 Promoter methylation analysis for TMPRSS2

The protein expression and promoter methylation status of *TMPRSS2* in the PRAD patients of the TCGA‐PRAD was explored through the UALCAN (University of Alabama Cancer) database. The association between the *TMPRSS2* expression and DNA methylation of the *TMPRSS2* promoter in the normal and PRAD tissues was conducted by the database of DNMIVD (DNA methylation interactive visualization database) (http://119.3.41.228/dnmivd/query_gene/?gene=TMPRSS2&panel=DMG&cancer=PRAD).^27^

### 2.5 Survival analysis for PRAD in TMPRSS2 expressions

Two expression groups based on the value of fragments per kilobase of exon model per million reads mapped (FPKM) in each gene in cancer patients were classified, and the correlations between expression level and patient survival were evaluated for PRAD cohort by GEPIA 2 (http://gepia2.cancer‐pku.cn/#survival) in TCGA and plotted a Kaplan‐Meier curve.^22^, ^26^, ^28^

### 2.6 Analysis for functional enrichment

The data of GO (Gene Ontology) and KEGG (Kyoto Encyclopedia of Genes and Genomes) pathway of the co‐expressed genes were analysed via the Enrichr database (https://maayanlab.cloud/Enrichr/enrich?dataset=5df6eaa47475293efe5b1514669a05bc#).^29^ The *P*‐value < .05 was set as a cut‐off criterion. The GEPIA 2 database was used to provide a group of genes with a similar expression pattern between TMPRSS2 and PRAD based on the TCGA‐PRAD cohort data.

## 3 RESULTS

### 3.1 Determination of TMPRSS2 conservation and expression in normal tissues

**FIGURE 1:**
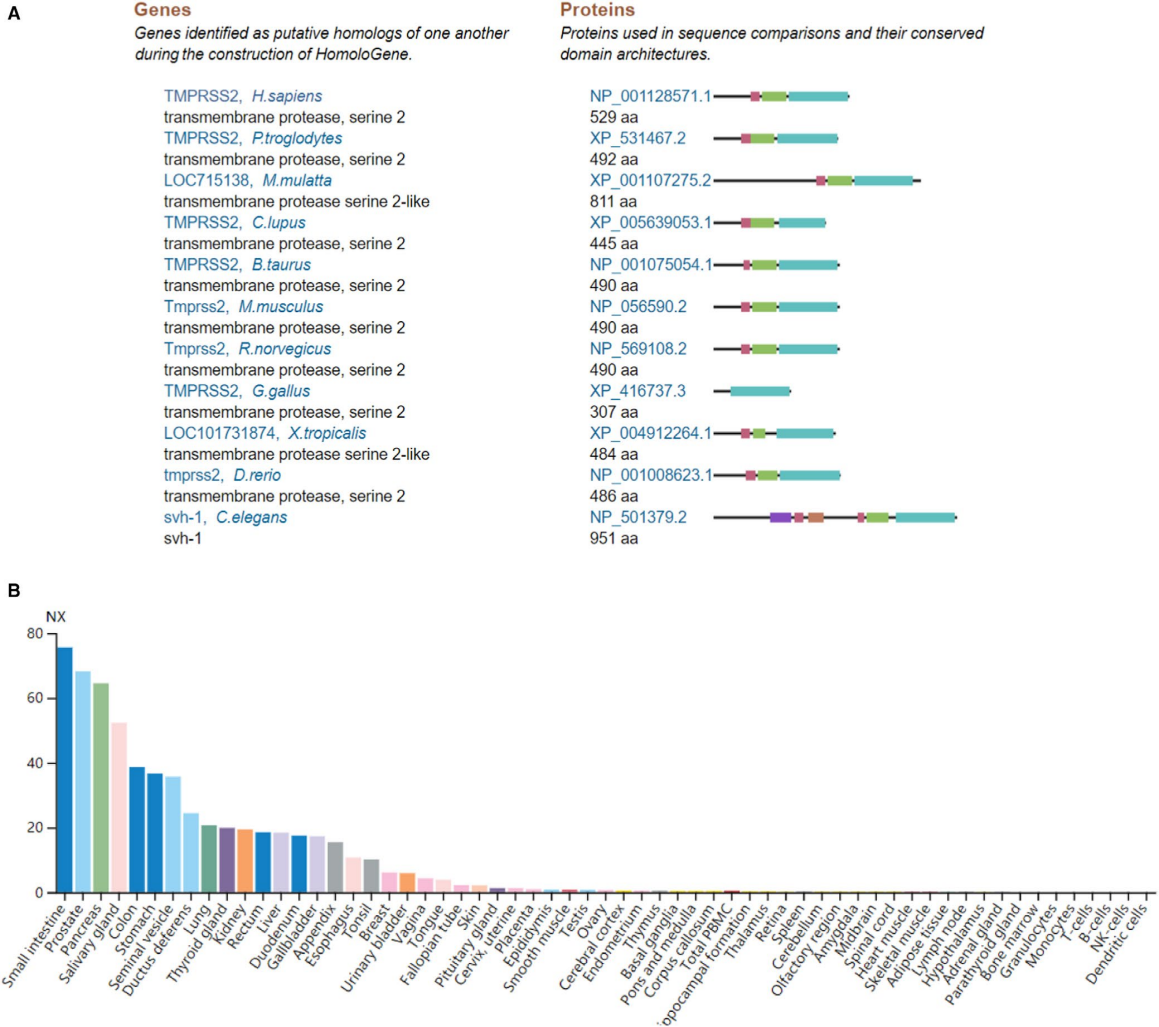
Homologs of the TMPRSS2 proteins and its expression in normal tissues and cells. A, Conservation for TMPRSS2 in eleven of different species. B, *TMPRSS2* mRNA expression in normal tissues. RNA expression overview shows RNA of consensus NX (Normalized eXpression) levels from 55 types of tissues and 6 types of blood cells, created by combining three different transcriptomics sources: RNA‐seq data from HPA, RNA‐seq data from GTEx and CAGE data from FANTOM5. Colour‐coding is based on tissue groups with common functional features. HPA, Human Protein Atlas. GTEx, Genotype‐Tissue Expression

Homologs of the TMPRSS2 protein showed that it is highly conserved in different species, including chimpanzee, Rhesus monkey, dog, cow, mouse, rat, chicken, zebra fish, *C*. *elegans*, and frog, with a trypsin‐like serine protease domain (Tryp_SPc, cd00190) (Figure 1A). Trypsin‐like serine protease is synthesized from inactive precursor zymogens by cleavage to generate their active forms. These suggest that TMPRSS2 from these different animals would potentially have enzymatic activity, making these species SARS‐CoV‐2’s probable natural hosts.

The expression profiles for *TMPRSS2* mRNA in humans were conducted from the data of RNA‐sequencing in the indicated fifty‐five types of tissues and six types of blood cells that are the consensus dataset from HPA, GTEx and FANTOM5. The RPKM values for *TMPRSS2* expression in the small intestine were found to be highest at 75.6, followed by the prostate (68.2), pancreas (64.5), salivary gland (52.3), colon (38.7), stomach (36.7), and lungs is ninth highest expression (20.7). The hypothalamus was found to be lowest with approximately 0.1 (Figure 1B). No expression was found in 6 blood cell types. Thus, these results demonstrated the biased expression profiles for *TMPRSS2* mRNA in the small intestine, prostate, lung and other tissues.

### 3.2 Expression analysis results of the *TMPRSS2* gene in prostate adenocarcinoma (PRAD)

**FIGURE 2:**
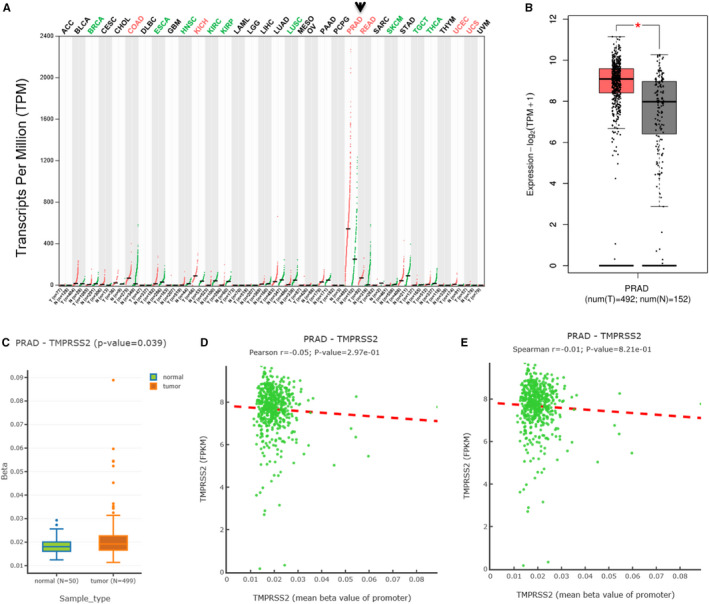
*TMPRSS2* expression and its promoter methylation status in tumour tissues of prostate adenocarcinoma (PRAD) and corresponding normal tissues. A, Expression profile for *TMPRSS2* in 32 different tumour tissues and their corresponding normal tissues (TCGA normal and GTEx data). Tissue‐wise expression using profiles. B, Expression profile for *TMPRSS2* in PRAD tumour tissues and the corresponding normal tissues (TCGA normal and GTEx data) (*: *P* <.01). Tissue‐wise expression using box plots. C, The promoter methylation status for the regulating *TMPRSS2* expression from PRAD. D, Pearson analysis for correlation between the mRNA expression and the methylation status for *TMPRSS2* from PRAD. E, Spearman analysis for correlation between the mRNA expression and the methylation status for the *TMPRSS2* gene from PRAD

Gene expression profile for *TMPRSS2* in 32 different tumour tissues and their corresponding normal tissues (TCGA normal and GTEx data) revealed six significantly up‐regulated (Figure 2A, in red colours) and six down‐regulated (Figure 2A, in green colours) in different types of adenocarcinomas. Importantly, both prostate adenocarcinoma and corresponding normal prostate tissues were highly expressed (Figure 2A, arrow). Further analysis in PRAD found that *TMPRSS2* expression is significantly up‐regulated (Figure 2B,*P* < .01).

To further know whether methylation modification affects *TMPRSS2* expression, the DNMIVD database was used to determine the promoter methylation status for TMPRSS2 in PRAD. However, the promoter methylation statuses for the TMPRSS2 in PRAD tissues were slightly increased in comparison with those of normal tissues (Figure 2C). Furthermore, the analysis of Spearman and Pearson correlations revealed a negative correlation between the *TMPRSS2* mRNA expression and its promoter methylation status for PRAD tissues (Figure 2D and F). Thus, promoter methylation of TMPRSS2 may not be the molecular mechanism for TMPRSS2 overexpression in PRAD tumours and PRAD's pathogenesis.

### 3.3 Analysis of isoform usage and isoform structures for TMPRSS2

**FIGURE 3:**
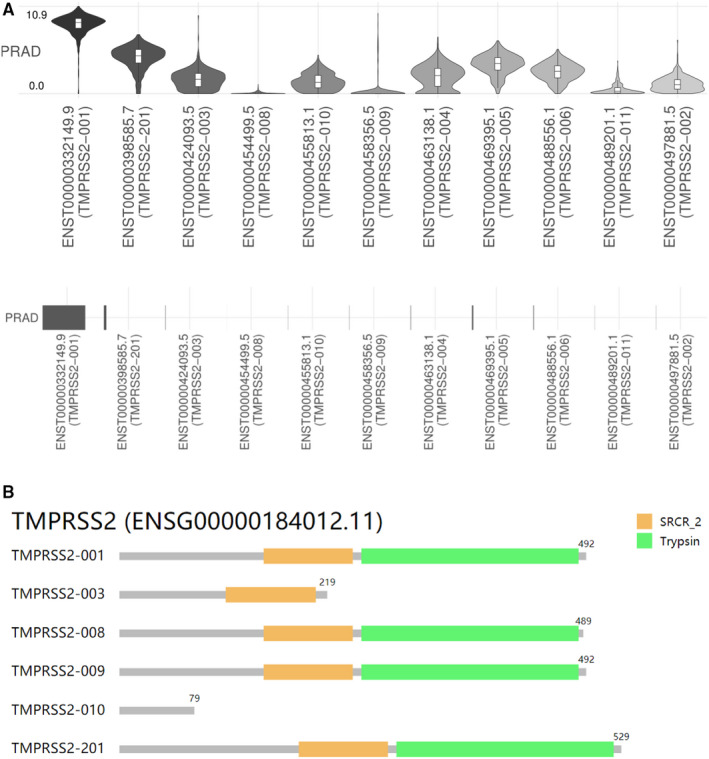
Isoform usage and isoform structures for TMPRSS2. A, Isoform usage for TMPRSS2. In this panel, the profiles for the expression distribution (violin plot, upper panel) and isoform usage (bar plot, lower panel) of TMPRSS2 in PRAD are presented. X: isoforms, Y: cancer type (PRAD). B, Isoform structures for TMPRSS2. Multiple isoforms and their protein domain structures are shown in an interactive plot. Note: 5 isoforms' information is missing from Figure 3A: TMPRSS2‐004, TMPRSS2‐005, TMPRSS2‐006, TMPRSS2‐011, TMPRSS2‐002. Trysin, Tryp_SPc domain

Different isoforms of SARS‐CoV‐2 receptors or entry proteins, for example isoforms of ACE2 expressed in the airway epithelium, may differentially contribute to host susceptibility to SARS‐CoV‐2 infection.^30^ Thus, to understand the expression of isoform usage and isoform structures for TMPRSS2 in PRAD tissues, we performed analysis of the GEPIA2 database, and the results are shown in Figure 3. From Figure 3, we found that eleven isoforms are expressed and used in PRAD tissues, with TMPRSS2‐001 as the highest, followed by TMPRSS2‐201, and TMPRSS2‐008 is the lowest (Figure 3A). Further isoform structures prediction showed that TMPRSS2‐001, TMPRSS2‐008, TMPRSS2‐009, and TMPRSS2‐201 have both SRCR_2 and Trypsin (Tryp_SPc) domains (Figure 3B). But TMPRSS2‐003 lacks Trypsin (Tryp_SPc), whereas TMPRSS2‐010 lacks neither SRCR_2 nor Trypsin (Tryp_SPc) (Figure 3B). Note that five of isoforms' information is missing, including ENST00000463138.1 (TMPRSS2‐004), ENST00000469395.1 (TMPRSS2‐005), ENST00000488556.1 (TMPRSS2‐006), ENST00000489201.1 (TMPRSS2‐011), and ENST00000497881.5 (TMPRSS2‐002). Altogether, these data suggest that TMPRSS2, with its high expression and usage of TMPRSS2‐001 and TMPRSS2‐201 containing both SRCR_2 and Trypsin (Tryp_SPc) in PRAD, should play important roles in tumorigenesis and COVID‐19 viral entry into PRAD tumour tissues.

### 3.4 Survival analysis for PRAD patients based on *TMPRSS2* expression

**FIGURE 4:**
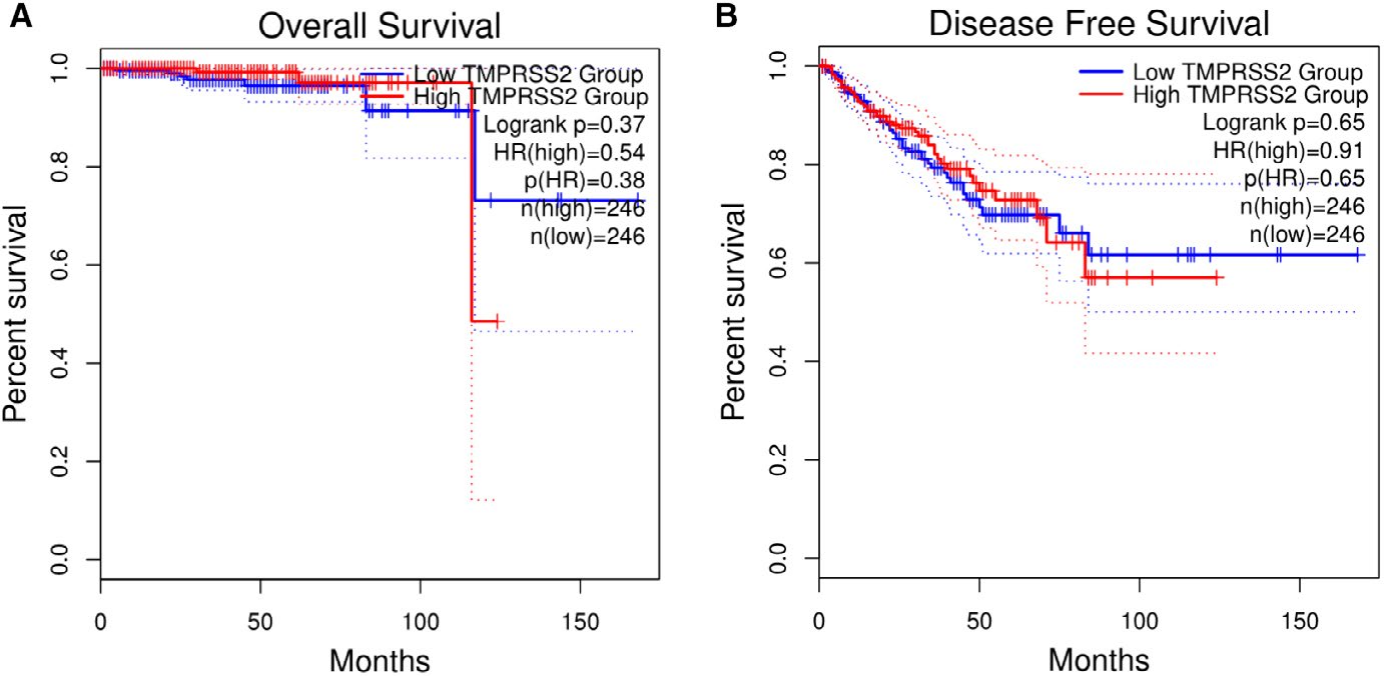
Survival analysis for the gene of *TMPRSS2* from PRAD patients for overall survival (A) and disease‐free survival (B). The GENT2 databases are used to assess on the TCGA‐COAD cohort data and plot Kaplan‐Meier curves

Given that the study focused on the expression of *TMPRSS2* and showed that the expression of *TMPRSS2* is higher in both normal and cancerous tissues from PRAD, clinical relationship between *TMPRSS2* expression and survival outcomes was also examined. The GENT2 databases were used to assess the TCGA‐COAD cohort data and plotted Kaplan‐Meier curves. The results are shown in Figure 4. From Figure 4, we found that high expression of *TMPRSS2* is not correlated with long survival in either overall survival (Figure 4A,*P* = .38) or disease‐free survival states (Figure 4B,*P* = .65). Thus, high expression of *TMPRSS2* may not be favourable for PRAD patient's survival.

### 3.5 Function analysis of co‐expressed genes for *TMPRSS2* in prostate cancer

**FIGURE 5:**
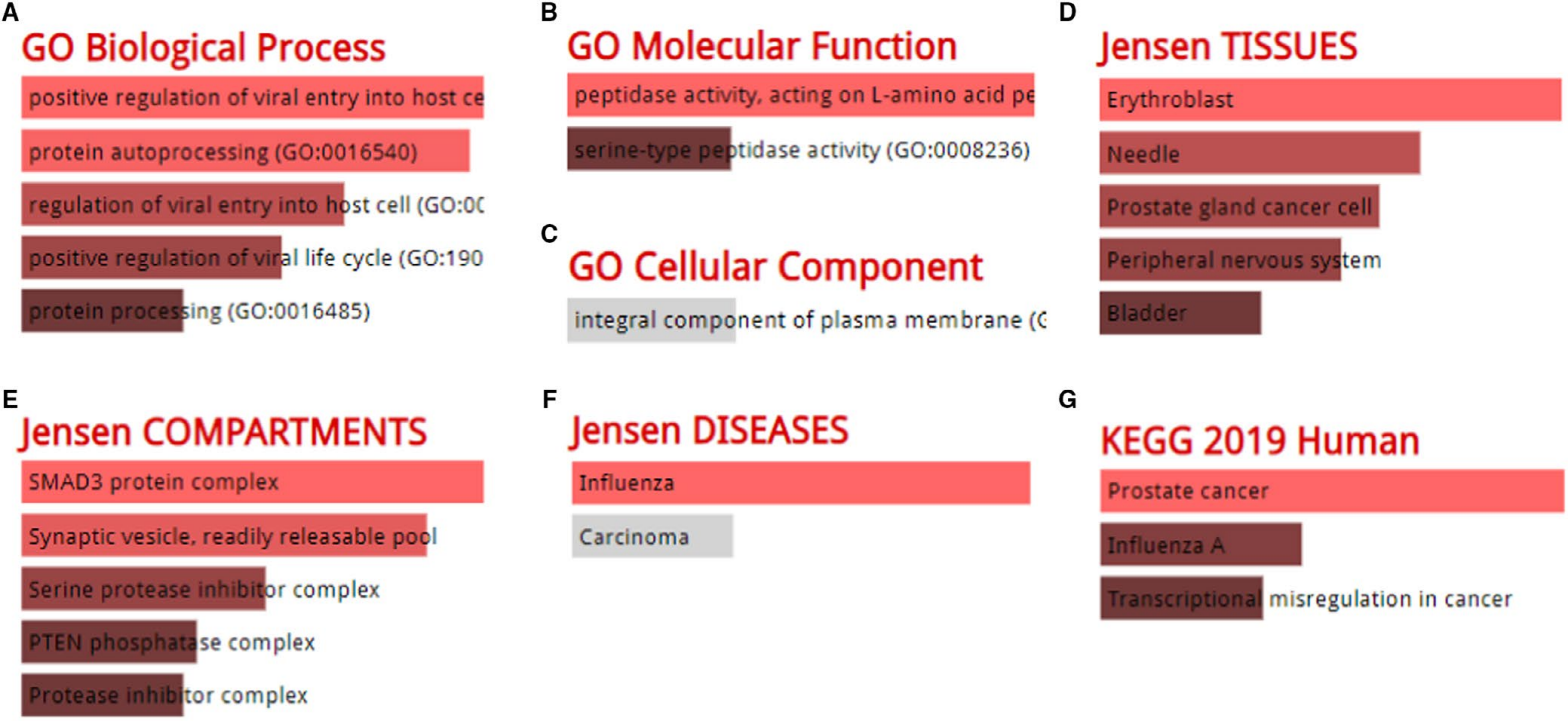
GO enrichment analysis results. The enriched information for biological process (A), molecular function (B), cellular component (C), associations between gene TMPRSS2 and tissues (D), associations between gene TMPRSS2 and cellular compartments (E), associations between gene TMPRSS2 and human disease (F) in GO analysis, and KEGG pathway (G) were obtained from the database of Enrichr, based on the TMPRSS2‐correlated genes. GO, Gene Ontology; KEGG, Kyoto Encyclopedia of Genes and Genomes

Analysis for co‐expression in GEPIA 2 database gave a total of 100 associated genes for *TMPRSS2* in PRAD (Supplementary Table S1). The GO analysis results from the Enrichr database are shown in Figure 5A‐F, and the data showed that we identified the related biological process (protein autoprocessing, protein processing, positive regulation of viral entry into host cell, positive regulation of viral life cycle), molecular function (peptidase activity acting on L‐amino acid peptides, serine‐type peptidase activity), cellular component (integral component of plasma membrane), Jensen TISSUES for associations of gene and tissues (erythroblast, needle, prostate gland cancer cell, peripheral nervous system, bladder), Jensen COMPARTMENTS for associations of gene and cellular components (SMAD3 protein complex, synaptic vesicle of readily releasable pool, serine protease inhibitor complex, PTEN phosphatase complex, protease inhibitor complex), and Jensen DISEASES for associations of gene and human diseases (influenza, carcinoma). KEGG pathway enrichment analysis from the Enrichr database further exploited three enriched pathways: prostate cancer, influenza A and transcriptional misregulation in cancer (Figure 5G). Thus, all these data demonstrated that the TMPRSS2 is mostly enriched in the regulation of viral entry, protein processing, serine‐type peptidase activity, prostate gland cancer cell expression, different complex(es) formation, diseases including influenza and carcinoma, and misregulation of pathways in prostate cancer, influenza A, and transcription in cancer.

## 4 DISCUSSION

Highly expressed entry proteins for SARS‐CoV‐2 may play critical roles for viral infection.^22^, ^31^, ^32^, ^33^ TMPRSS2‐expressing cell line has been reported to be highly susceptible to SARS‐CoV‐2 infection.^34^, ^35^ Thus, it is important to assess TMPRSS2 expression in normal and cancer tissues, particular in prostate cancer tissues, for help in predicting the cancer patients’ susceptibility to SARS‐CoV‐2 infection and the disease outcome. In this study, by analysing NCBI database, HPA datasets and GEPIA 2 databases, we found that *TMPRSS2* is highly conserved from different species and highly expressed in normal human tissues, including the small intestine, prostate, pancreas, salivary gland, colon, stomach, seminal vesicle and lung. It is also increased in PRAD cancer tissues, indicating that SARS‐CoV‐2 might attack not only the lungs and other normal organs, but also PRAD cancer tissues. Thus, TMPRSS2‐mediated actions should explain the low fatality of prepubertal children and the differences between sexes by viral entry.^36^, ^37^ By analysing the expression of isoform usage and isoform structures for TMPRSS2 in PRAD tissues, we found that TMPRSS2 expressed and used eleven isoforms in PRAD tissues, with isoform TMPRSS2‐001 as the highest, followed by TMPRSS2‐201. Further isoform structures prediction showed that these two highly expressed isoforms have both SRCR_2 and Trypsin (Tryp_SPc, cd00190) domains. Tryp_SPc is a catalytic triad for serine proteases, and SRCR_2 is a scavenger receptor cysteine‐rich domain, which is essential for TMPRSS2 functional roles, suggesting that high expression of TMPRSS2‐001 and TMPRSS2‐201 with both SRCR_2 and Trypsin (Tryp_SPc) domains in PRAD tissues should play important roles for tumorigenesis and entry for SARS‐CoV‐2 in PRAD patients. This is supported by Montopoli *et al* from Italy that prostate cancer patients do have an increased risk of SARS‐CoV‐2 infections compared to non‐cancer patients.^38^ Then, we performed a series for functional annotation and enrichment analyses in TMPRSS2, demonstrating that TMPRSS2 is mostly enriched in regulation of viral entry into host cell, protein processing, and serine‐type peptidase activity, and is associated with prostate gland cancer cell expression, different complex(es) formation, human diseases of influenza and carcinoma, and pathways in prostate cancer, influenza A, and transcription misregulation in cancer. Altogether, even though high expression of TMPRSS2 may not be favourable for PRAD patient's survival, increased TMPRSS2 expression in these patients should play a role in susceptibility for the SARS‐CoV‐2 viral infection and clinical severity for COVID‐19 symptoms.

To further understand the mechanism of how methylation modification affects TMPRSS2 expression, methylation analysis of TMPRSS2 promoter in PRAD revealed that the methylation of the TMPRSS2 promoter in PRAD is slightly increased compared to that in normal tissue, implying that hypomethylation of TMPRSS2 promoter may not be the mechanism for TMPRSS2 overexpression in PRAD tumour tissues and PRAD's pathogenesis. Androgen‐induced TMPRRSS2 gene expression may be one of regulatory mechanisms.^3^, ^39^ But other regulatory mechanisms would also be exists, so further study should be conducted.

In conclusion, the *TMPRSS2* gene is highly expressed in normal prostate tissues and increased significantly in PRAD cancer tumours, indicating the susceptibility for the SARS‐CoV‐2 infection and high severity of COVID‐19 symptoms. Our study highlights the value of protecting PRAD patients by targeting or androgen‐mediated therapeutic strategies in the COVID‐19 pandemic.^36^, ^40^

## 5 ETHICS APPROVAL

The study has the Ethical Committee approval granted by the Southwest Medical University. This article does not contain any studies with human participants performed by any of the authors.

## CONFLICTS OF INTEREST

None.

## AUTHOR CONTRIBUTION

**Jingliang Cheng:** Investigation (equal); Software (equal). **Ju Zhou:** Validation (equal). **Shangyi Fu:** Writing‐original draft (equal); Writing‐review & editing (equal). **Jiewen Fu:** Formal analysis (equal); Investigation (equal). **Baixv Zhou:** Investigation (equal). **Hanchun Chen:** Project administration (equal). **JUNJIANG FU:** Conceptualization (equal); Formal analysis (equal); Funding acquisition (equal); Project administration (equal); Supervision (equal); Writing‐original draft (equal); Writing‐review & editing (equal). **Chunli Wei:** Investigation (equal); Project administration (equal).

## Supporting information

Table S1

## Data Availability

Data sharing is not applicable to this article as no new data were analyzed in this study.
